# Cognitive and Psychosocial Development in Young Children with Brain Tumors: Observations from a Clinical Sample †

**DOI:** 10.3390/children6110128

**Published:** 2019-11-19

**Authors:** Niki Jurbergs, Jennifer L. Harman, Ansley E. Kenney, Katherine Semenkovich, Andrew E. Molnar, Victoria W. Willard

**Affiliations:** 1 Department of Psychology, St. Jude Children’s Research Hospital, 262 Danny Thomas Place, Memphis, TN 38105, USA; niki.jurbergs@stjude.org (N.J.); jennifer.harman@stjude.org (J.L.H.); ansley.kenney@nationwidechildrens.org (A.E.K.); ksmnkvch@memphis.edu (K.S.);; 2 Department of Psychology, University of Memphis, Memphis, TN 38152, USA

**Keywords:** pediatric brain tumor, young children, cognitive functioning, psychosocial functioning, early childhood, childhood cancer

## Abstract

Survivors of pediatric brain tumor (BT) are known to be at risk for developing cognitive and psychosocial late effects. Young age at treatment (≤6 years) is typically considered to put patients at increased risk. However, there is limited research specifically exploring functioning in these young patients. Cognitive and psychosocial data were retrospectively abstracted from medical charts for 79 young patients (54.4% male) treated for BT with a variety of treatment modalities (e.g., surgery, radiation therapy, chemotherapy). Children were clinically assessed at 4.52 years of age (range = 1.48–5.98) and most were off-therapy (74.4%). Mean performances on developmental (68.3 ± 10.02), cognitive (88.09 ± 18.38), and pre-academic (86.84 ± 19.75) measures were all below average. Parent report of adaptive functioning was also below average (82.10 ± 16.21), but psychosocial functioning was generally within normal limits. Most patients had impaired functioning (scores <10th percentile) in at least one domain assessed. Exploratory analyses revealed that many patients (27.3–60.6%) exhibited a significant discrepancy between domains of cognitive functioning (e.g., verbal and spatial). Young children treated for BT experienced high rates of impairment in cognitive, pre-academic, and adaptive domains. Future work is needed to focus on serial longitudinal assessment of these young patients, as well as dedicated intervention and prevention efforts.

## 1. Introduction

Pediatric patients with brain tumors are at high risk for cognitive and psychosocial weaknesses as a result of their disease and the neurotoxic therapies (e.g., surgery, radiation therapy, chemotherapy) required to treat it [1]. Those patients who are youngest at diagnosis and treatment (less than 6 years of age) are at increased risk due to the rapid brain development that also occurs at this time [2,3,4]. Unfortunately, despite a significant research literature documenting neuropsychological weaknesses (e.g., deficits in working memory, processing speed, attention, executive functioning) in patients with brain tumors, explicit focus on and assessment of those diagnosed when very young is somewhat limited.

The early childhood years mark a time of rapid brain development and skill acquisition. For children with brain tumors whose long-term neuropsychological weaknesses are marked by difficulties acquiring new skills at the same rates as their peers [5], the diagnosis and treatment during this age range (<6 years of age) can be potentially devastating. Given these youth experience a significant neurological hit (e.g., presence of a brain tumor, surgery to remove, receipt of neurotoxic therapies) before entering school, understanding their early cognitive and psychosocial profile would allow for the development of effective early interventions that may prevent the emergence of later concerns (e.g., learning problems, reduced graduation rates). Unfortunately, the limited research that has been done with patients diagnosed at an early age has primarily documented the presence of impairments when these patients are school-aged (age 8 or older) and several years post-diagnosis and treatment [6,7]. There are few, if any, studies that explicitly evaluate the neurocognitive and psychosocial abilities of patients with brain tumors during early childhood (e.g., ages 0 to 6). As such, there remains a significant gap in our understanding of the early cognitive and psychological functioning of young patients with brain tumors around the time of diagnosis and treatment, and prior to school entry.

Fay-McClymont and colleagues [8] recently documented the neuropsychological functioning of a small sample of young children diagnosed with medulloblastoma before 6 years of age, treated with high-dose chemotherapy, and assessed between 5 and 7 years of age. Results indicated that most children had low average to average global cognitive functioning, though a quarter of the sample had clinically significant impairment (functioning at <10th percentile) in at least one domain assessed [8]. Given the possibility that these patients will continue to fall further behind as they age [5], these children are likely to be at a noted disadvantage as they enter formal schooling and will require significant intervention and accommodation.

While the work by Fay-McClymont and colleagues [8] provides some initial evidence regarding the early cognitive functioning of young patients with brain tumors, the paper was limited to those with medulloblastoma treated with high-dose chemotherapy. As there are numerous other tumor types (e.g., ependymoma, glioma) and treatment modalities (e.g., surgery, radiation therapy), additional work is needed. Further, the study focused on children 5 to 7 years of age and did not include evaluation results from when children were younger (<5 years). As such, the objective of the current paper was to describe the cognitive and psychosocial profiles of young children treated for brain tumors and assessed in a hospital-based psychology clinic. All patients were younger than 6 years of age at both diagnosis/treatment and psychological evaluation. Given past research, it was hypothesized that, on average, patients would demonstrate overall functioning that was below average. However, significant variability was also predicted, such that many patients would exhibit a significant impairment (performance at <10th percentile) in at least one domain of functioning.

## 2. Materials and Methods

### 2.1. Procedures

The Institutional Review Board approved this project as a retrospective review of clinical records (IRB number XPD16-107, Pro00006253). Subsequent to review and approval, data from psychological assessments completed in a hospital-based Psychology Clinic between 1 July 2010 and 30 June 2015 were abstracted from the medical record. During this period, 79 pediatric patients with brain tumors under the age of 6 completed an assessment that included at least one measure evaluating one or more of the following domains: Cognitive/developmental, adaptive, academic, and/or psychosocial functioning. All patients were under 6 years of age at the time of assessment, and all were clinically referred. While specific referral reasons are not available for each patient, given the known risk for cognitive late effects in this population [1], it is likely that a proportion of the sample was referred for evaluation as standard of care. Other patients may have been referred due to parent or provider concern for developmental delay or learning concerns, or for evaluation prior to school entry/re-entry.

Assessment information, key demographic (age, gender), and medical (e.g., age at diagnosis, treatment history) information were abstracted from the medical record. As all assessment batteries were developed to answer a clinical question, there was no standard battery administered. As such, when possible, assessment data were collapsed across similar measures and domains (e.g., adaptive behavior, full-scale IQ, internalizing problems) so as to provide the largest available sample size. Subscales presented were limited to those that were available for the majority of measures (see Measures for more information).

### 2.2. Measures

Developmental functioning: Two measures of developmental functioning, the Mullen Scales of Early Learning [9] and the Bayley Scales of Infant Development, 3rd edition [10], were administered to 10 patients (6 Mullen, 4 Bayley). These measures are designed to assess the early functioning of very young children (<5 years) and include subscales assessing gross and fine motor skills and receptive and expressive language, as well as an overall composite. The composite score was expressed as a standard score (M = 100, SD = 15), while subscales were converted to scaled scores (M = 10, SD = 3) for ease of interpretation. Common indices across both measures included a composite, as well as gross motor, fine motor, receptive language, and expressive language subscales.

Cognitive functioning: The majority of children were administered measures of cognitive functioning, with 61 children administered at least part of one of the following measures: Differential Abilities Scale, 2nd edition [11] (n = 39), Wechsler Preschool and Primary Scale of Intelligence, 3rd edition [12] (n = 16) or 4th edition [13] (n = 4), and the Woodcock Johnson Tests of Cognitive Abilities, 3rd edition [14] (n = 2). Common indices across measures included full-scale IQ (all measures), as well as verbal (all measures), nonverbal (all measures), and spatial (DAS-II, WPPSI-IV only) composites. All scores are standard scores (M = 100, SD = 15). 

Academic functioning: More than two-thirds of patients (n = 55, 69.6%) were administered the School Readiness Composite of the Bracken Basic Concept Scale, 3rd edition [15]. This composite assesses recognition of pre-academic concepts such as colors, numbers, letters, shapes, and size comparisons, and is expressed as a standard score (M = 100, SD = 15).

Adaptive functioning: One of two parent-report measures of adaptive functioning were completed for 70 (88.6%) patients. Seventeen parents completed the Vineland Adaptive Behavior Scales, 2nd edition [16] and 53 completed the Adaptive Behavior Assessment System, 2nd edition [17]. Common indices across the two measures include an adaptive behavior composite, as well as indicators of socialization and practical/daily living skills. All scores are standard scores (M = 100, SD = 15).

Psychosocial functioning: One of two parent-report measures of emotional/behavioral functioning were completed for 66 (83.5%) patients. Eleven parents completed the Child Behavior Checklist [18] and 55 parents completed the Behavioral Assessment System for Children, 2nd edition [19]. Three common and relevant scores—internalizing, externalizing, and attention problems—were collapsed across the two measures. Scores are expressed as T-scores (M = 50, SD = 10), with higher scores indicating more problems.

### 2.3. Analytical Plan

Given differences in assessment strategies by age, the decision was made to analyze data from children administered developmental measures (n = 10) and children administered cognitive measures (n = 67) separately (2 patients were administered parent-report measures only). Parent-report measures were analyzed for the group as a whole. 

Descriptive statistics were used to characterize the sample for each domain, including mean, SD, and range of scores. To add clinical context to the scores, descriptive values based on Wechsler’s nomenclature [12,13] (SS ≤ 69 extremely low, 70–79 borderline, 80–89 low average, 90–109 average, 110–119 high average, 120–129 superior, ≥130 very superior) were used in narrative descriptions. For each domain of functioning, the percentage of patients falling within the impaired range was calculated. Specifically, standard scores <80 and/or 1.3 SD above/below the mean and/or below the 10th percentile as relevant to the individual measure were deemed “impaired.” This score range is consistent with the borderline range or below, as well as other literature [8,20]. 

One sample *t*-tests were then used to compare our sample to the normative mean on each measure. Independent samples *t*-tests, ANOVAs, and linear regression were used to assess differences in functioning based on clinically relevant factors (e.g., sex, treatment status, treatment with radiation therapy, age at diagnosis).

## 3. Results

### 3.1. Participants

The final sample included 79 pediatric patients with brain tumors who were, on average, 4.52 years old at assessment (SD = 1.20, range 1.48–5.98 years). The sample was 54.4% (n = 43) male and 68.4% (n = 54) white. The majority of patients (75.9%, n = 60) were off-therapy at the time of assessment, with a mean time off-treatment of 1.55 years (SD = 1.29, range 0–5.17 years). Common diagnoses included medulloblastoma (16.5%), astrocytoma (15.2%), ependymoma (13.9%), and optic glioma (10.1%). For all demographic and treatment variables, please see Table 1.

### 3.2. Developmental Functioning

Patients administered developmental measures were, on average, 2.29 years of age (SD = 0.82) at assessment. Half were off-therapy at the time of assessment, and two had received radiation therapy. Broadly, these patients demonstrated significant weaknesses, with overall composite scores ranging from the extremely low to the low average range (Table 2). Nine of 10 patients fell within the impaired range (standard scores < 80). Subscale scores were also quite low, with 50% to 90% of patients falling within the impaired range (scaled scores ≤ 5) across domains. 

### 3.3. Cognitive and Academic Functioning

**Figure 1:**
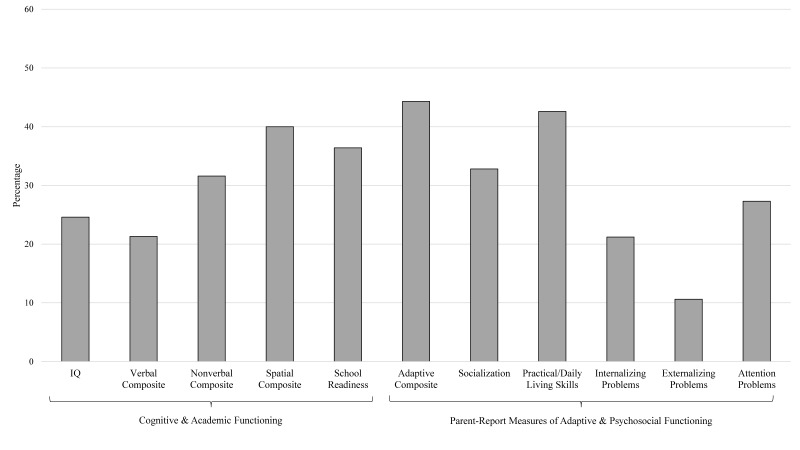
Percentage of sample administered an objective measure of cognitive or pre-academic functioning (n = 65), a parent-reported measure of adaptive (n = 70), or psychosocial functioning (n = 66) that fell within the impaired range (cognitive/academic, adaptive: Standard Score < 80; psychosocial: T-score ≥ 64).

On average, patients administered cognitive and/or academic assessments (n = 67) were 4.88 years of age (SD = 0.82). Broadly, mean performance across cognitive domains was in the low average range (with the exception of the verbal composite, which was in the average range) and all composites were significantly below the normative mean (Table 3). The percentage of patients in the impaired range (SS < 80) varied from 21.3% (verbal composite) to 40% (spatial composite) (Figure 1). Mean performance on the school readiness composite was also in the low average range, with 36.4% of patients performing in the impaired range. Overall, 46.3% of patients administered an objective assessment of cognitive or academic functioning were in the impaired range in at least one domain, with 32.8% in the impaired range in at least two domains. 

### 3.4. Parent-Reported Adaptive and Psychosocial Functioning

Mean ratings on measures of adaptive and psychosocial functioning for the entire sample are in Table 4. Adaptive functioning was generally within the low average range, with more than 30% of patients in the impaired range (SS < 80) in each domain (Figure 1). In contrast, mean ratings of psychosocial functioning were in the average range. One-sample t-tests revealed that mean scores for internalizing and attention problems were statistically above normative means; the mean for externalizing problems was consistent with the normative mean. Notably, 27.3% of patients were in the impaired range (T ≥ 64) for attention problems. Overall, 73.3% of patients administered a parent-report measure were in the impaired range in at least one domain assessed, with 48% in the impaired range in at least two domains.

### 3.5. Predictors of Functioning

A series of independent sample *t*-tests, ANOVAs, and linear regression were used to assess the impact of relevant demographic and medical factors on domains of functioning. There were no differences based on gender, treatment status at time of assessment, treatment with radiation therapy, type of radiation therapy (focal vs. craniospinal, photon vs. proton), hearing loss, or history of posterior fossa syndrome for any measure (all *p*’s > 0.08). However, age at diagnosis and time since diagnosis were both significant predictors of the cognitive composite, though not adaptive functioning. Specifically, patients who were younger at diagnosis (*β* = 0.30, *t* = 2.34, *p* < 0.03) and further from diagnosis at the time of assessment (*β* = −0.39, *t* = −3.13, *p* < 0.01) demonstrated worse cognitive functioning.

### 3.6. Exploratory Analyses

Upon further examination of individual cognitive scores, it was observed that there were frequently large discrepancies between domains within individual patient’s performances. As such, we explored this further using the measure of cognitive functioning administered most frequently. The DAS-II Upper Early Years edition [11] was administered to 33 patients and includes three composite scores that make up the full-scale IQ equivalent. Discrepancies between composites ranged from 1 to 51 standard score points (mean = 15.71, SD = 11.82, median = 14.50). The spatial composite was most frequently the weakest composite score (n = 22), while the verbal and nonverbal composites were most frequently the strongest ability (both n = 14).

The DAS-II manual [11] provides guidance on the size of discrepancies between composite scores that are significant at the *p* < 0.01 level, and indicate that the overall global composite (e.g., IQ) may not be valid due to significant variability. Nine of 33 patients (27.3%) demonstrated discrepancies between the verbal and nonverbal reasoning composites that were greater than this marker (≥19 points). Further, 20 patients (60.6%) demonstrated discrepancies between the verbal and spatial composites (≥15 points) and 16 (48.5%) between the spatial and nonverbal reasoning composites (≥17 points). Finally, 17 patients (51.5%) demonstrated significant discrepancies in more than one area (e.g., verbal–spatial and verbal–nonverbal reasoning).

## 4. Discussion

This paper sought to describe the cognitive and psychosocial functioning of children under six years of age with a brain tumor. Given the significant risk of deficits associated with young age at diagnosis, and the paucity of research on patients in this age range, we conducted a retrospective review of patients seen in the psychology clinic associated with a cancer center. As hypothesized, the overall functioning of our sample was below average across most domains assessed—cognitive, pre-academic, adaptive—though psychosocial functioning was generally within normal limits, even if mean scores were statistically different than normative means (e.g., internalizing and attention problems). Rates of impairment across domains were notable, with a significant proportion of patients in the impaired range (<10th percentile) across domains. However, in contrast to expectations and other studies [7,8], medical and demographic factors were largely unrelated to variability in functioning. The exception was younger age at diagnosis and longer time since diagnosis, which were both associated with worse performance on the cognitive composite.

Findings from exploratory analyses were striking in that the majority of patients demonstrated significant variability between domains of cognitive functioning—verbal, nonverbal, spatial—that were large enough to invalidate the use of an overall cognitive composite/indicator of IQ. While the use of IQ composites has been previously questioned due to the high rates of deficits in working memory and processing speed in the pediatric brain tumor population [21,22], this analysis highlights the variability in broader cognitive abilities as well. This finding is consistent with our clinical observations over 20 years of administering assessments that variability across domains of cognitive functioning is relatively common, but the research literature is mixed on this finding. Specifically, in a study of older survivors of pediatric brain tumors (ages 6 to 16), Kahalley and colleagues [22] demonstrated relatively evenly developed verbal and nonverbal abilities, while Bonner and colleagues documented discrepancies in verbal and nonverbal functioning in survivors aged 6 to 16 [23]. It is possible that the more uneven development of cognitive abilities documented in this study is a reflection of the young age at which our patients were both treated and assessed. Future research is necessary to more explicitly examine and document this finding in a prospective and longitudinal fashion.

While the cognitive and psychosocial risks of a brain tumor diagnosis are well known, this study highlights the early and significant impairments experienced by children who are diagnosed and treated before they truly engage in formal schooling. With almost half of patients demonstrating a clinically significant impairment in cognitive functioning, these children are urgently in need of intervention efforts. It is likely that many of these patients will require formal academic accommodations via special education services [24], and that intervention may need to be intensified over time. 

While intervention efforts for off-therapy patients are certainly necessary, these young patients may uniquely benefit from prevention efforts—or interventions and services delivered while receiving treatment (a quarter of our sample was on therapy at the time of assessment). Patients under three years of age—those that were particularly impaired in this sample—may be eligible for state-funded early intervention services made possible through Part C of the Individuals with Disabilities Education Act [25]. A diagnosis of cancer makes children eligible for these services in many states, and in those states where cancer is not an automatically qualifying condition, there may be ways for parents or professionals to advocate for these essential services [26]. For slightly older children (i.e., ages three to six years), the development of prevention and intervention programs that target social interaction, early learning skills, and overall functioning should be a high priority. Unfortunately, these children are often too young to be eligible for hospital-based school services, and, due to treatment, are not gaining exposure to peers and other non-home or hospital environments, such as daycare or preschools. These experiences are critical for both cognitive and psychosocial development [27,28].

Along with intervention efforts, additional research is needed to systematically characterize the longitudinal trajectory of cognitive and psychosocial functioning of pediatric patients after the diagnosis of a brain tumor in very early childhood (<6 years of age). Indeed, the current paper represents just a preliminary snapshot of functioning at a single point in time. Given that there were largely no differences in functioning based on traditional risk factors such as treatment with radiation therapy or being on versus off therapy at the time of assessment, questions remain regarding whether there may be differences in risk factors and trajectories for patients with brain tumors diagnosed before and after six years of age. Unfortunately, until standardized serial assessments are completed with patients in this specific age range, these questions cannot be adequately answered. 

This paper is not without limitations. Perhaps most importantly, data for this study were captured from the medical charts of pediatric patients referred for clinical assessment in a cancer center psychology clinic. As such, there are likely biases in the sample that completed assessments as compared to those that were not referred. However, given the known cognitive and psychosocial risks associated with a brain tumor diagnosis [1], this is a population that is frequently referred for assessment as a standard of care [29]. Relatedly, given the clinical nature of these assessments, a standardized battery was not used and analyses required the collapsing of domains of functioning across measures. This also meant that some domains of functioning that are known to be impacted in youth with brain tumors—working memory, executive functioning, processing speed—were not captured at all. Of note, this is in part a function of measurement as there are limited objective assessments of these domains available for young children. 

Ultimately, findings from this paper highlight the significant and impairing consequences of a diagnosis and treatment of a brain tumor in early childhood. While young age at diagnosis has long been recognized as a risk factor for late cognitive and psychosocial effects, the current paper joins with other recent efforts [8] in establishing the more acute effects of a brain tumor diagnosis in young childhood. Additional focus on assessment and intervention efforts for this vulnerable age group is sorely needed.

## Figures and Tables

**Table 1:** Demographic and medical information (n = 79).

		N (%)	M ± SD, Range (Years)
Age			4.52 ± 1.20, 1.48–5.98
Gender			
Male		43 (54.4)	
Female		36 (45.6)	
Race			
White		54 (68.4)	
Black		16 (20.3)	
Other		9 (11.4)	
Common Diagnoses			
Medulloblastoma		13 (16.5)	
Astrocytoma		12 (15.2)	
Ependymoma		11 (13.9)	
Optic Glioma		8 (10.1)	
Age at Diagnosis			2.39 ± 1.46, 0.00–5.75
Treatment Status			
On		20 (25.3)	
Off		59 (74.7)	
Time off-treatment			1.58 ± 1.28, 0.08–5.17
Treatment			
Surgery		65 (82.3)	
Chemotherapy		56 (70.9)	
Radiation Therapy		48 (60.8)	
Parameters	Focal	28 (58.3)	
	CSI + Focal	20 (41.7)	
Type	Proton	9 (18.8)	
	Photon	39 (81.2)	
Relapse/Progression		14 (17.7)	
Posterior Fossa Syndrome		12 (15.2)	
Hearing Loss		22 (27.8)	
Seizures		19 (24.1)	

CSI—craniospinal irradiation.

**Table 2:** Functioning of young children on measures of developmental functioning (n = 10).

	Mean ± SD	Range	*t*	*p*	N (%) Impaired
Age at Assessment	2.29 ± 0.82	1.48–3.99			
Developmental Testing					
Composite ^a^	68.3 ± 10.02	55–85	−10.01	<0.001	9 (90.0)
Gross Motor ^b^	3.6 ± 2.56	1–7	−7.04	<0.001	6 (75.0)
Fine Motor ^b^	4.1 ± 3.69	1–11	−5.05	0.001	7 (70.0)
Receptive Language ^b^	4.1 ± 3.07	1–11	−6.08	<0.001	8 (80.0)
Expressive Language ^b^	5.2 ± 1.87	3–8	−8.10	<0.001	5 (50.0)

^a^ Standard score: M = 100, SD = 15, impaired < 80; ^b^ scaled score: M = 10, SD = 3, impaired ≤ 5.

**Table 3:** Cognitive and academic functioning.

	N	Mean ± SD	Range	*t*	*p*	N (%) Impaired
Age at Assessment	67	4.88 ± 0.81	2.52–5.98			
Cognitive Functioning						
FSIQ	57	88.09 ± 18.38	43–139	−4.89	<0.001	14 (24.6)
Verbal Composite	61	91.43 ± 16.45	53–134	−4.07	<0.001	13 (21.3)
Nonverbal Composite	57	89.28 ± 18.87	45–140	−4.29	<0.001	18 (31.6)
Spatial Composite	35	83.63 ± 20.33	42–121	−4.76	<0.001	14 (40.0)
Academic Functioning						
School Readiness Composite	55	86.84 ± 19.75	40–126	−4.94	<0.001	20 (36.4)

Standard Score (M = 100, SD = 15), Impaired < 80.

**Table 4:** Parent-reported adaptive and psychosocial functioning.

	N	Mean ± SD	Range	*t*	*p*	N (%) Impaired
Adaptive Functioning ^a^						
Composite	70	82.10 ± 16.21	50–120	−9.34	<0.001	31 (44.3)
Socialization	67	88.09 ± 17.14	49–124	−5.69	<0.001	22 (32.8)
Practical/Daily Living Skills	68	82.69 ± 16.04	45–114	−8.90	<0.001	29 (42.6)
Psychosocial Functioning ^b^						
Internalizing Problems	66	54.02 ± 11.09	33–84	2.94	0.005	14 (21.2)
Externalizing Problems	66	50.67 ± 11.60	32–95	0.47	0.64	7 (10.6)
Attention Problems	66	56.29 ± 10.24	34–75	4.99	<0.001	18 (27.3)

^a^ Standard Score (M = 100, SD = 15), Impaired < 80; ^b^ T-score (M = 50, SD = 10), Impaired ≥ 64.
